# Quantum structural fluxion in superconducting lanthanum polyhydride

**DOI:** 10.1038/s41467-023-37295-1

**Published:** 2023-03-25

**Authors:** Hui Wang, Pascal T. Salzbrenner, Ion Errea, Feng Peng, Ziheng Lu, Hanyu Liu, Li Zhu, Chris J. Pickard, Yansun Yao

**Affiliations:** 1 Key Laboratory for Photonic and Electronic Bandgap Materials (Ministry of Education), School of Physics and Electronic Engineering, Harbin Normal University, 150025 Harbin, China; 2 International Center for Computational Method & Software, College of Physics, Jilin University, 130012 Changchun, China; 3 Department of Materials Science & Metallurgy, University of Cambridge, 27 Charles Babbage Road, Cambridge, CB3 0FS UK; 4 Fisika Aplikatua Saila, Gipuzkoako Ingeniaritza Eskola, University of the Basque Country (UPV/EHU), Europa Plaza 1, 20018 Donostia/San Sebastián, Spain; 5 Centro de Física de Materiales (CSIC-UPV/EHU), Manuel de Lardizabal Pasealekua 5, 20018 Donostia/San Sebastián, Spain; 6 Donostia International Physics Center (DIPC), Manuel de Lardizabal Pasealekua 4, 20018 Donostia/San Sebastián, Spain; 7 College of Physics and Electronic Information, Luoyang Normal University, 471022 Luoyang, P. R. China; 8 State Key Laboratory of Superhard Materials and International Center of Future Science, Jilin University, 130012 Changchun, China; 9 Department of Physics, Rutgers University, Newark, NJ 07102 USA; 10 Advanced Institute for Materials Research, Tohoku University 2-1-1 Katahira, Aoba, Sendai 980-8577 Japan; 11 Department of Physics and Engineering Physics, University of Saskatchewan, Saskatoon, Saskatchewan S7N 5E2 Canada

**Keywords:** Structure of solids and liquids, Superconducting properties and materials, Electronic properties and materials

## Abstract

The discovery of 250-kelvin superconducting lanthanum polyhydride under high pressure marked a significant advance toward the realization of a room‐temperature superconductor. X-ray diffraction (XRD) studies reveal a nonstoichiometric LaH_9.6_ or LaH_10±δ_ polyhydride responsible for the superconductivity, which in the literature is commonly treated as LaH_10_ without accounting for stoichiometric defects. Here, we discover significant nuclear quantum effects (NQE) in this polyhydride, and demonstrate that a minor amount of stoichiometric defects will cause quantum proton diffusion in the otherwise rigid lanthanum lattice in the ground state. The diffusion coefficient reaches ~10^−7^ cm^2^/s in LaH_9.63_ at 150 gigapascals and 240 kelvin, approaching the upper bound value of interstitial hydrides at comparable temperatures. A puzzling phenomenon observed in previous experiments, the positive pressure dependence of the superconducting critical temperature *T*_c_ below 150 gigapascals, is explained by a modulation of the electronic structure due to a premature distortion of the hydrogen lattice in this quantum fluxional structure upon decompression, and resulting changes of the electron-phonon coupling. This finding suggests the coexistence of the quantum proton fluxion and hydrogen-induced superconductivity in this lanthanum polyhydride, and leads to an understanding of the structural nature and superconductivity of nonstoichiomectric hydrogen-rich materials.

## Abstract (web summary)

The role of stoichiometric defects in the superconducting polyhydride LaH_10±δ_ has received little attention so far. Here, the authors use molecular-dynamics simulations to show that a small amount of stoichiometric defects will cause quantum proton diffusion in the otherwise rigid lanthanum lattice.

## Introduction

Superconductivity at near room temperature has been discovered in clathrate polyhydrides at megabar pressures^1–7^. Determining the crystal structure responsible for the superconductivity is of critical importance, yet a great challenge^8,9^. Experimentally, difficulties in probing lighter elements arise in XRD, impeding a direct determination of the complete lattice symmetry. The measured *T*_c_ and its pressure dependence (d*T*_c_/d*p*) tighten constraints on structural models; however, calculations on the candidate structures based on Bardeen–Cooper–Schrieffer (BCS) theory^10^ unequivocally suggest a negative d*T*_c_/d*p*^11–20^, which contradicts the experimentally observed positive d*T*_c_/d*p* in some pressure regimes (Supplementary Fig. 1). This phenomenon was initially observed in the 250-kelvin superconducting lanthanum polyhydride^1^, and later in polyhydrides of yttrium^6,7^ and calcium^21^ with *T*_c_ reaching 257 K and 212 K, respectively. This ‘positive d*T*_c_/d*p* contradiction’ presents a major obstacle to our full understanding of the crystal structures of these superconducting polyhydrides.

As the first superconductor with a *T*_c_ above 250 K, lanthanum polyhydride has been studied by several groups independently^1–5^. In addition to the high *T*_c_, a positive d*T*_c_/d*p* was observed between 137 and 150 GPa by measurements on four samples (samples 1–4 in Ref. ^1^) synthesized at different pressures with laser heating. XRD measurements determine that the lanthanum atoms form a face-centered cubic (*fcc*) lattice at pressures of 137–218 GPa, while the hydrogen atoms have undetermined locations within the lattice. Based on the measured crystal volume, the hydrogen-to-lanthanum (H/La) ratio around the maximum *T*_c_ was estimated to be 9.6 (150 GPa)^1^ or 9–11 (180–200 GPa)^2,3^ in the two studies, respectively. This trend was recently confirmed by new experiments on the *fcc* → *C*2/*m* phase transformation at *p*_c_ = 135 GPa, which shows an even steeper decrease of *T*_c_ below *p*_c_^5^.

With a moderate synthetic pressure and high symmetry, ‘*fcc*’ lanthanum polyhydride provides a model system for exploring the structural nature of high-*T*_c_ hydrides in both experiment and theory. In fact, ab initio calculations have guided the experimental discovery of lanthanum polyhydrides, and predicted the appearance of high *T*_c_ superconductivity, i.e., an estimated *T*_*c*_ of 280-kelvin for *fcc*-LaH_10_ at 210 GPa^11,12^. This prediction turned out to be very close to the *fcc* lanthanum polyhydride later synthesized, with some differences in *T*_c_, synthetic pressure, and hydrogen content. Recently, it has been theorized that the inclusion of quantum atomic fluctuation is essential for a correct calculation of *T*_c_ and the pressure boundary of *fcc*-LaH_10_, which is dictated by the quantum nature of lanthanum polyhydride structures^16^. However, a negative d*T*_c_/d*p* predicted at 137–150 GPa^16^ remains in contradiction to the experimental observations (Supplementary Fig. 1). This disagreement suggests that critical factors have been overlooked in previous calculations of superconductivity. In particular, the stoichiometric defect observed by experimental studies may play an important role in determining the structure and underlying superconductivity.

## Results and discussion

**Fig. 1:**
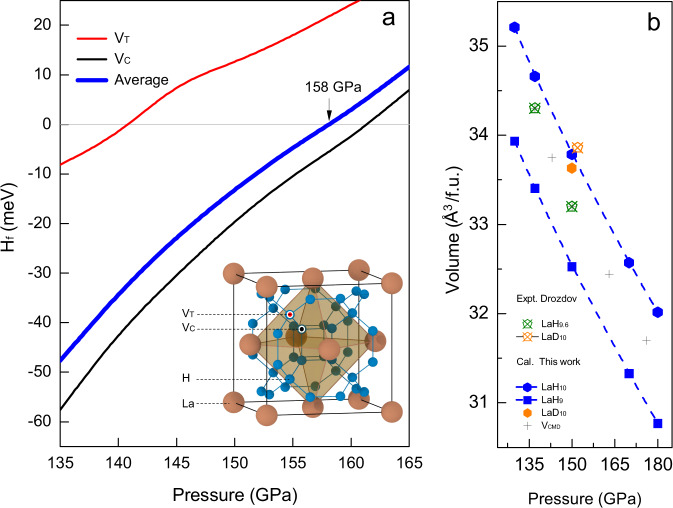
Vacancy formation enthalpy and pressure-volume relation. **a** The formation enthalpy (H_f_) of a single vacancy in *fcc*-LaH_10_ at two inequivalent lattice sites in the clathrate hydrogen framework, *V*_T_ and *V*_C_, and their configurational average calculated at different pressures using 2 × 2 × 2 extension of conventional unit cell of *fcc*-LaH_10_. The locations of *V*_T_ and *V*_C_ are illustrated in a conventional unit cell of the *fcc*-LaH_10_ structure by red and black balls, respectively. **b** The experimental pressure-volume relations of *fcc*-LaH_9.6_ and *fcc*-LaD_10_ measured by Drozdov et al.^1^ compared to the theoretical values of *fcc*-LaH_10_, *fcc*-LaH_9_ and *fcc*-LaD_10_ derived from quantum simulations at constant pressure and temperature of 300 K. The volumes selected for subsequent quantum simulation (*V*_CMD_) for *fcc*-LaH_9.63_ are thereby linked to the ‘quantum’ pressure. Formula unit is abbreviated as f.u.

Although minor in amount, defects in solid materials can strongly affect their properties. The thermodynamic stability of a defect in high-pressure solids as well as the relative stabilities of different defect structures can be evaluated by the defect formation enthalpy (H^f^). As shown in Fig. 1a, the crystal structure of *fcc*-LaH_10_ is represented as the insertion of an ‘H cube’ into octahedral interstices of the fluorite-LaH_2_ structure. In this model, we calculated the H^f^ for a vacancy defect either at a corner of the H cube (*V*_C_) or at a tetrahedral interstice of the La lattice (*V*_T_), and found that the configurationally averaged H^f^ becomes negative below 158 GPa. This suggests that *fcc*-LaH_10_ is prone to vacancy defects, which agrees well with the experimental finding of hypostoichiometric LaH_10-δ_ at 150 GPa specifically LaH_9.6_^1^. Notably, the calculated ‘vacancy occurring region’ coincides the region where the *T*_c_ of the *fcc* lanthanum polyhydride depends positively on the pressure (which previous calculations have failed to predict), indicating that the vacancy structure has a key role to play in the superconductivity.

A low concentration of vacancies in hydrogen sites does not change the XRD pattern of the *fcc* lanthanum polyhydride, which is determined primarily by the La sublattice. However, these vacancies significantly affect the dynamics of hydrogen in the crystal (Supplementary Fig. 2). The distortion in the hydrogen sublattice spreads out away from the vacancy sites in both the *V*_*C*_ and *V*_T_ models of LaH_10_ with an H/La ratio of 9.97, and even results in a ‘liquid-like’ H framework in the former at 150 GPa. For LaH_10-δ_, the presence of vacancies changes the potential energy surface, while quantum nuclear fluctuation is expected to affect the transport behavior of vacancies. The calculated zero-point energy (ZPE) of ~150 meV/LaH_10_ is comparable to the vacancy migration energy of 160/200 meV for the migration path of *V*_C_ → *V*_C’_/*V*_C_ → *V*_T_ at 150 GPa. This suggests that nuclear zero-point motion can promote proton hopping to neighboring vacancies and cause the LaH_10-δ_ ground state to not be a single structure around which the atoms vibrate, but instead one where the protons dynamically explore different vacancies in a fixed *fcc*-La framework.

We refer to this behavior as being ‘fluxional’ in the sense used by Goncharov et al. for phase IV of solid hydrogen^22^. It is well-established by experiment and theory that thermal effects dominate the fluxionality in this structure: A classically ordered phase III replaces the fluxional phase IV at low temperature^23–25^. Here, we demonstrate that, in the presence of vacancies, hydrogen NQEs are strong enough—and indeed essential—to stabilize a fluxional framework in the *fcc* lanthanum polyhydride. Therefore, we are dealing with a quantum fluxional structure (QFS).

**Fig. 2:**
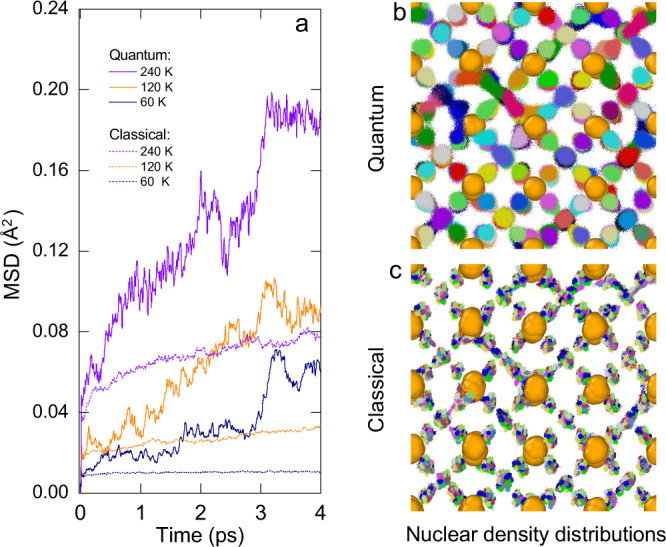
Structural fluxion in LaH_9.63_ at 150 GPa. **a** The proton MSD derived from centroid trajectories of the CMD simulations and those of MD simulations. **b** The [100] view of the quantum nuclear density distribution at 60 K extracted from a CMD simulation, with full consideration of the 16 beads. Neighboring protons are illustrated in different colors. **c** the [100] view of the classical nuclear density distribution in 16 MD simulation runs of 4 ps distinguished by various proton colors. The 16 runs were initialized from different centroid configurations of the 4-picosecond CMD trajectory with a sampling interval of 0.25 ps.

Taking LaH_9.63_ as an example, we investigate the NQE and thermal effects on the structural fluxion at 150 GPa and low temperatures up to 240 K. The stoichiometry is selected according to the experimental estimation (LaH_9.6_). Our theoretical simulations suggest a H/La ratio of 9.54 (or 9.71) for the experimental samples synthesized at 150 GPa (or 137 GPa), in good agreement with experiments^1^ (Fig. 1b). We adopt ab initio centroid molecular dynamics (CMD)^26^ to treat the nuclei quantum mechanically. As shown by the mean square displacement (MSD) curves in Fig. 2a, the simulations reveal appreciable proton migration, with equivalent mean migration distances of vacancies reaching 1.0 to 2.0 Å between 60 and 240 K (Supplementary Fig. 3). These distances are almost as large as or larger than the shortest H-H separation around 1.2 Å. This is consistent with the ‘network’-like density distribution patterns obtained, for instance, at 60 K (Fig. 2b). In contrast, the diffusivity is much smaller in ab initio molecular dynamics (MD), where the nuclei are treated classically (Fig. 2a, c). This confirms that the structural fluxion in LaH_9.63_ is dominated by the NQE, rather than thermal effects. The crucial role of vacancies in this intriguing quantum phenomenon is demonstrated by a comparison to the dynamics of stoichiometric *fcc*-LaH_10_ at the same pressure, which exhibits no diffusion below 700 K^27^. Experimentally, the occurrence of ‘quenched-in’ vacancies in polyhydrides is inevitable, as a result of the thermal treatment of the samples. This finding therefore suggests that vacancy effects interact with the strong NQE to result in strong quantum structural fluxion in polyhydrides.

**Fig. 3:**
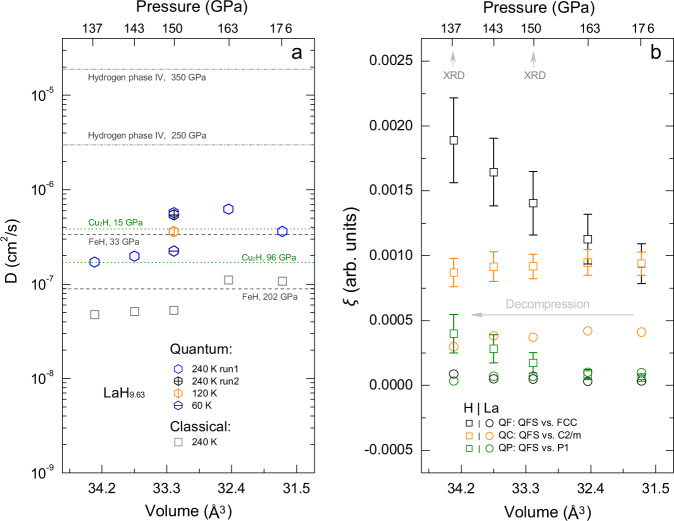
Pressure effects on the diffusivity and structural distortions. **a** The proton diffusion coefficient *D* derived from the MSD in CMD simulations (run1) of 4 ps, and from the average MSD of four MD simulations of 24 ps. The *D* in three additional CMD simulations of 4 ps at 150 GPa are also shown. These are two simulations at 120 K and 60 K, and a simulation using higher total-energy convergence criteria (run2). The MSD in the CMD simulations is derived from the centroid trajectories. The measured *D* in Cu_2_H^29^ and FeH^31^, and calculated *D* in phase IV hydrogen^30^ are shown for comparison. **b** The configurational distance *ξ* (with error bar indicating the standard deviation) of QFS from the crystal lattices of static *fcc* and *C*2*m* phases, and a triclinic *P*1 structure for H and La substructure. The *P*1 structure is built by scaling the lattice parameter a of a QFS sampled from the CMD simulation of LaH_9.63_ at 176 GPa.

We approximate the proton diffusion coefficient *D* in LaH_9.63_ from the slope of the MSD in simulations at 240 K and between 137 to 176 GPa. The temperature is chosen to be close to the measured *T*_c_ of the *fcc* lanthanum polyhydride. As shown in Fig. 3a, the *D* value falls in the range of 10^−6^ to 10^−7^ cm^2^/s in our quantum simulation, whereas it is significantly lower in the classical simulation. The *D* value is 2–3 orders of magnitude below the threshold criterion for classical superionicity (~10^−4^ cm^2^/s) for freely diffusing protons^28^. This notwithstanding, it reaches or indeed exceeds the upper bound diffusivities observed in interstitial hydrides at room temperature, such as 3.8 × 10^−7^ cm^2^/s in *fcc*-Cu_2_H^29^, and approaches that of phase IV hydrogen^30^. With increasing pressure, *D* decreases in interstitial hydrides (e.g. Cu_2_H^29^ and FeH^31^), due to the contraction of the metal lattice and the resulting increase of the activation energy for proton hopping. In phase IV hydrogen, on the other hand, *D* increases with pressure^30^, probably owing to the drastic increase of the proton hopping rate with the shortened of the distance. Our preliminary results suggest that the unique ‘host-guest’ structure of LaH_9.63_ likely induces a significant competition between the two mechanisms mentioned, resulting in a nonmonotonic pressure trend of the coefficient *D*.

In LaH_9.63_, proton diffusion breaks the balance of internal Coulomb repulsions in the ‘H cube’ located in octahedral interstices of La sublattice, which therefore distorts the H sublattice. Lattice expansion promotes the distortion, as indicated by the heightening of the second coordination shell (peaked at ~1.85 Å) in the average radial distribution function [RDF, *g*(r), Supplementary Fig. 4], which is correlated to a smearing of the inter- and inner-cube H-H separations. Based on a configurational distance (denoted as *ξ*) of crystal fingerprint matrices^32^, which is sensitive to local structural changes, we parametrized the structural difference of the QFS relative to the crystal lattices of static *fcc* and *C*2/*m* phases, and a triclinic *P*1 structure mimicking the QFS, as shown in Fig. 3b by *ξ*_qf_, *ξ*_qc_ and *ξ*_qp_ respectively. For the H substructure, we find *ξ*_qf_ > *ξ*_qc_ > *ξ*_qp_, with the *ξ*_qf_ much beyond the other two at larger volumes, revealing an increasing distortion of the H sublattice in QFS from *fcc* to lower symmetry upon decompression. For the La substructure, *ξ*_qc_ > *ξ*_qf_ ≈ *ξ*_qp_, with *ξ*_qc_ much larger than *ξ*_qf_ and *ξ*_qp_. This agrees with the XRD measurements that determine an *fcc* sublattice for La atoms above 137 GPa^1^. The tiny distortions related to *ξ*_qf_ and *ξ*_qp_ are both within the uncertainty of refinements for the *fcc* phase in XRD studies (Supplementary Fig. 4). The results suggest a premature low-symmetry distortion of the H substructure upon volume expansion relative to the La substructure in the quantum fluxional LaH_9.63_.

**Fig. 4:**
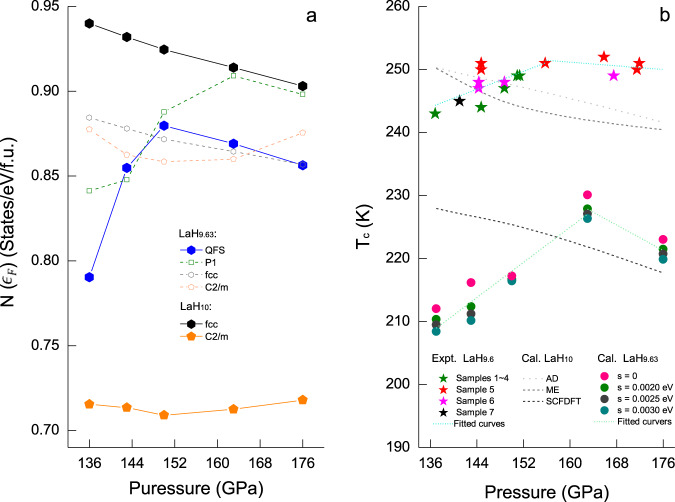
Pressure effects on electronic properties and superconductivity. **a** The pressure trend of the averaged electronic density of states at the Fermi level $N\left({\epsilon }_{F}\right)$ in quantum fluxional LaH_9.63_ (with distributions and standard deviation of statics shown in Supplementary Fig. 5), and the pressure trend of $N\left({\epsilon }_{F}\right)$ in static *P*1-LaH_9.63_, *fcc*-LaH_10_, and *C*2/*m*-LaH_10_. The $N\left({\epsilon }_{F}\right)$ of LaH_9.63_ in *fcc* and *C*2/*m* phase estimated using the rigid-band approximation are also shown for comparison. **b** The pressure trend of *T*_c_ in quantum fluxional LaH_9.63_ calculated based on various Gaussian smearings (σ) of the phonon spectrum $F\left(\omega \right)$, together with the values measured for LaH_9.6_ by Drozdov et al.^1^ and those derived from AD, Migdal–Eliashberg equations (ME), and superconducting DFT (SCDFT) for *fcc*-LaH_10_ by Errea et al.^16^. Fitted lines are shown to guide the sight.

Using the centroid configurations of CMD simulations at 240 K, i.e. the QFS, we calculated the electronic density of states at the Fermi level $N\left({\epsilon }_{F}\right)$ in LaH_9.63_, which exhibits opposite pressure trends at pressure below and above 150 GPa (Fig. 4a). The *fcc*-LaH_10_ (quantum) crystal has a monotonously negative pressure dependence of $N\left({\epsilon }_{F}\right)$ above 100 GPa^12,16^, and this prediction can be extended to LaH_9.63_ using the rigid-band model of the electronic structure by virtue of an artificial shift of ${\epsilon }_{F}$^33^. The pressure dependences of $N\left({\epsilon }_{F}\right)$ in the *C*2/*m* phase of LaH_10_ and LaH_9.63_ (within the rigid-band approximation) are both non-monotonic, similar to quantum fluxional LaH_9.63_, suggesting that pressure effects on d$N\left({\epsilon }_{F}\right)$/d*p* are much more significant in distorted structures compared to the high-symmetry *fcc* phase. The non-monotonic pressure trend of $N\left({\epsilon }_{F}\right)$ in LaH_9.63_ is a statistical consequence of the QFS having an average *fcc*-La sublattice with diversely distorted H substructures. This trend cannot be attributed to a single classical configuration, but can roughly be reproduced by the *P*1 structure mimicking the QFS (Supplementary Fig. 5), which implies that the premature distortion of the H substructure (e.g. *fcc* → *P*1 symmetry) accounts for the sign change of d$N\left({\epsilon }_{F}\right)$/d*p* in the quantum fluxional LaH_9.63_.

The analytic McMillan^34^ and Allen-Dynes (AD)^35^ formulas (see details in Supplemental Information) have played an important role in analyzing the mechanism of pressure-dependent superconductivity in high-*T*_c_ hydrides. In these formulas, the electron-phonon coupling (EPC) constant *λ* depends explicitly on characteristic parameters of both electrons and phonons, in addition to the average of the electron-phonon matrix elements, $\langle {{\rm I}}^{2}\rangle$. Specifically, *λ* can be expressed as the product of $\langle {{\rm I}}^{2}\rangle /M$ (denoted as *β*, with $M$ being atomic mass) and $N\left({\epsilon }_{F}\right)/\left({\bar{\omega }}_{2}^{2}\right)$ (denoted as *ζ*, with ${\bar{\omega }}_{2}$ being the second frequency moment of the Eliashberg function). *ζ* directly describes the competition between electron and phonon contributions. Analysis of literature data suggest that *ζ* decreases more than two times faster than *β* increases upon compression (Supplementary Table 1). Furthermore, it was found that d*λ*/d*p* plays a dominating role in determining the d*T*_c_/d*p*, despite the fact that the *T*_c_ also depends explicitly on ${\omega }_{\log }$ and ${\bar{\omega }}_{2}$/${\omega }_{\log }$ (with ${\omega }_{\log }$ being the logarithmic frequency moment of the Eliashberg function). These findings suggest two rules for single crystal phases of high-*T*_c_ hydrides: (I) phonons affect d*T*_c_/d*p* mainly through the parameter *λ*; and (II) d*λ*/d*p* is primarily determined by *dζ*/d*p*, rather than *dβ*/d*p*.

In view of *ζ* being proportional to $N\left({\epsilon }_{F}\right)$, the pressure trend of $N\left({\epsilon }_{F}\right)$ in LaH_9.63_ (Fig. 4a) suggests a sign change of d*T*_c_/d*p* at studied pressures. Since the quantum fluxional nature of LaH_9.63_ forbids a direct calculation of the Eliashberg function ${\alpha \left(\omega \right)}^{2}F\left(\omega \right)$ by density functional methods, a quantitative confirmation of this is unachievable yet. However, considering that the change is so significant that even qualitative estimations could provide insights, we study the sign of d*T*_c_/d*p* slope in LaH_9.63_ under two approximations, as the first step to access the problem: (I) calculating ${\omega }_{\log }$ and ${\bar{\omega }}_{2}$ based on the phonon spectrum $F\left(\omega \right)$ of LaH_9.63_, combined with ${\alpha \left(\omega \right)}^{2}$ approximated by that of quantum *fcc*-LaH_10_; (II) approximating the *β* in LaH_9.63_ by that of the quantum *fcc*-LaH_10_. The rationale of such approximation is based on empirical rules, and the fact that quantum fluxional LaH_9.63_ largely retains the same local atomic environment as quantum *fcc*-LaH_10_ (Supplementary Fig. 6). The $F\left(\omega \right)$ derived from the Fourier transform of the velocity autocorrelation functions in the CMD simulations at 240 K, as well as the approximated ${\alpha \left(\omega \right)}^{2}$ and ${\alpha \left(\omega \right)}^{2}F\left(\omega \right)$ are shown in Supplementary Fig. 7.

With this approach, we evaluated the pressure trend of *T*_c_ in LaH_9.63_ using the AD formula (with parameters listed in Supplementary Table 2). It does indeed exhibit a positive d*T*_c_/d*p* slope at 137–163 GPa, in qualitative agreement with experimental observation^1^ (Fig. 4b). Moreover, a negative d*T*_c_/d*p* slope is obtained at higher pressures in line with both experimental observations and theoretical results for *fcc*-LaH_10_ (Supplementary Fig. 1). The standard deviation of $N\left({\epsilon }_{F}\right)$ (Supplementary Fig. 5) reveals fluctuations of the electron structure near the Fermi level, which may affect the *T*_c_ through parameters *ζ* and *λ*. However, considering that superconductivity of LaH_9.6_ is measured at a time scale far beyond that of the simulation, it is feasible to calculate the *T*_c_ by a averaged $N\left({\epsilon }_{F}\right)$. Recently, the *C*2/*m* phase has been successfully prepared at 120 GPa by abrupt decompression of the *fcc* phase^5^. A positive d*T*_c_/d*p* slope measured in the subsequent compression was attributed to a boost in the *T*_c_ due to phonon softening. As illustrated in the literature^12,16^, the softening of phonons results in a negative d*T*_c_/d*p* slope for *fcc*-LaH_10_, in contrast to the experimental observations below 150 GPa^1^. However, the concept of ‘a higher *T*_*c*_ near structural instability’^5^ agrees well with our finding of a premature distortion of hydrogen substructure relative to an average *fcc*-La substructure in quantum fluxional LaH_9.63_, upon decompression to 137 GPa.

In summary, the present work illustrates in lanthanum polyhydride the coexistence of quantum proton fluxion and hydrogen-induced high-*T*_*c*_ superconductivity. A premature distortion of hydrogen substructure upon volume expansion and its impact on the superconductivity through modulating the electronic structure is revealed. The findings support the experimental argument concerning the hypostoichiometric nature of the superconducting samples, reveal the crucial role of vacancy defects in the structure, and provide a theoretical explanation for the positive d*T*_c_/d*p* slope of lanthanum polyhydride, with implications for understanding the structure and superconductivity of other hydrogen-rich materials. With significant progress of nuclear magnetic resonance techniques in diamond anvil cells, the measurement of proton mobility in metal hydrides is currently accessible at megabar pressures^29,31^. We anticipate that continuous experimental and theoretical studies of quantum fluxional structure of high-*T*_c_ hydrides will stimulate new theoretical tools for understanding the superconducting behavior of quantum fluxional materials that cannot be precisely described by a single underlying static structure.

## Methods

The ab initio calculations were performed using the plane-wave pseudopotential method, as implemented in the Vienna ab initio simulation program (VASP)^36^, with the Perdew–Burke–Ernzerhof (PBE) Generalized Gradient Approximation (GGA) density functional^37^, and the bare ion Coulomb potential treated in the projector augmented wave (PAW) framework^38^. The vacancy formation enthalpy of *fcc*-LaH_10_ was calculated from vacancy structures of *fcc*-LaH_10_ and *B*_2_/*n*-H_2_^39^, with enthalpies calculated using the third-order Birch–Murnaghan isothermal equation of state (EOS)^40^ from ab initio energy-volume relations, as implemented in the EOS code^41^. The vacancy migration energy was calculated by the climbing image nudged elastic band method (CI-NEB)^42^. The quantum nuclear dynamics were studied by path-integral molecular dynamics (PIMD) and centroid molecular dynamics (CMD)^30^, as implement in the PIMD code^43^. The superconductivity was analyzed using McMillan’s theory^34^ with the *T*_c_ estimated by the Allen-Dynes equations^35^. The crystal structures and MD trajectories were visualized by the VESTA^44^ and OVITO softwares^45^ respectively. More details are shown in the Supplementary Information.

## Supplementary information

Supplementary Information

Peer Review File

## Data Availability

All data are available in the paper and from the author upon request.
