# Supplementary material for: Quantum structural fluxion in superconducting lanthanum polyhydride
Source: Nat Commun. 2023 Mar 25;14:1674. doi: 10.1038/s41467-023-37295-1 (PMC10039887; doi:10.1038/s41467-023-37295-1)
Supplement: Supplementary file 1 — Supplementary Information [file 41467_2023_37295_MOESM1_ESM.pdf]

Supplementary Information of

"Quantum structural fluxion in superconducting lanthanum polyhydride"

Hui Wang[1,2*], Pascal T. Salzbrenner[3], Ion Errea[4,5,6], Feng Peng[7], Ziheng Lu[3], Hanyu Liu[2,8], Li Zhu[9], Chris J. Pickard[3,10] and Yansun Yao[11]

[1]*Key Laboratory for Photonic and Electronic Bandgap Materials (Ministry of Education), School of Physics and Electronic Engineering, Harbin Normal University, Harbin 150025, China*

[2]*International Center for Computational Method & Software, College of Physics, Jilin University, Changchun 130012, China*

[3]*Department of Materials Science & Metallurgy, University of Cambridge, 27 Charles Babbage Road, Cambridge CB3 0FS, United Kingdom*

[4]*Fisika Aplikatua Saila, Gipuzkoako Ingeniaritza Eskola, University of the Basque Country (UPV/EHU), Europa Plaza 1, 20018 Donostia/San Sebastián, Spain*

[5]*Centro de Física de Materiales (CSIC-UPV/EHU), Manuel de Lardizabal Pasealekua 5, 20018 Donostia/San Sebastián, Spain*

[6]*Donostia International Physics Center (DIPC), Manuel de Lardizabal Pasealekua 4, 20018 Donostia/San Sebastián, Spain*

[7]*College of Physics and Electronic Information, Luoyang Normal University, Luoyang 471022, P. R. China*

[8]*State Key Laboratory of Superhard Materials and International Center of Future Science, Jilin University, Changchun 130012, China*

[9]*Department of Physics, Rutgers University, Newark, NJ 07102, USA*

[10]*Advanced Institute for Materials Research, Tohoku University 2-1-1 Katahira, Aoba, Sendai, 980-8577, Japan*

[11]*Department of Physics and Engineering Physics, University of Saskatchewan, Saskatoon, Saskatchewan S7N 5E2, Canada*

*e-mail: wh@fysik.cn

Table of contents

1. Extended Data Figures; Page 1 ~ 7

   Supplementary Figure 1 ~ Supplementary Figure 7

2. Extended Data Tables; Page 8 ~ 9

   Supplementary Table 1 ~ Supplementary Table 2

3. Computational Details; Page 10 ~ 15

4. Supplementary References; Page 16 ~ 17

Supplementary Figure 1

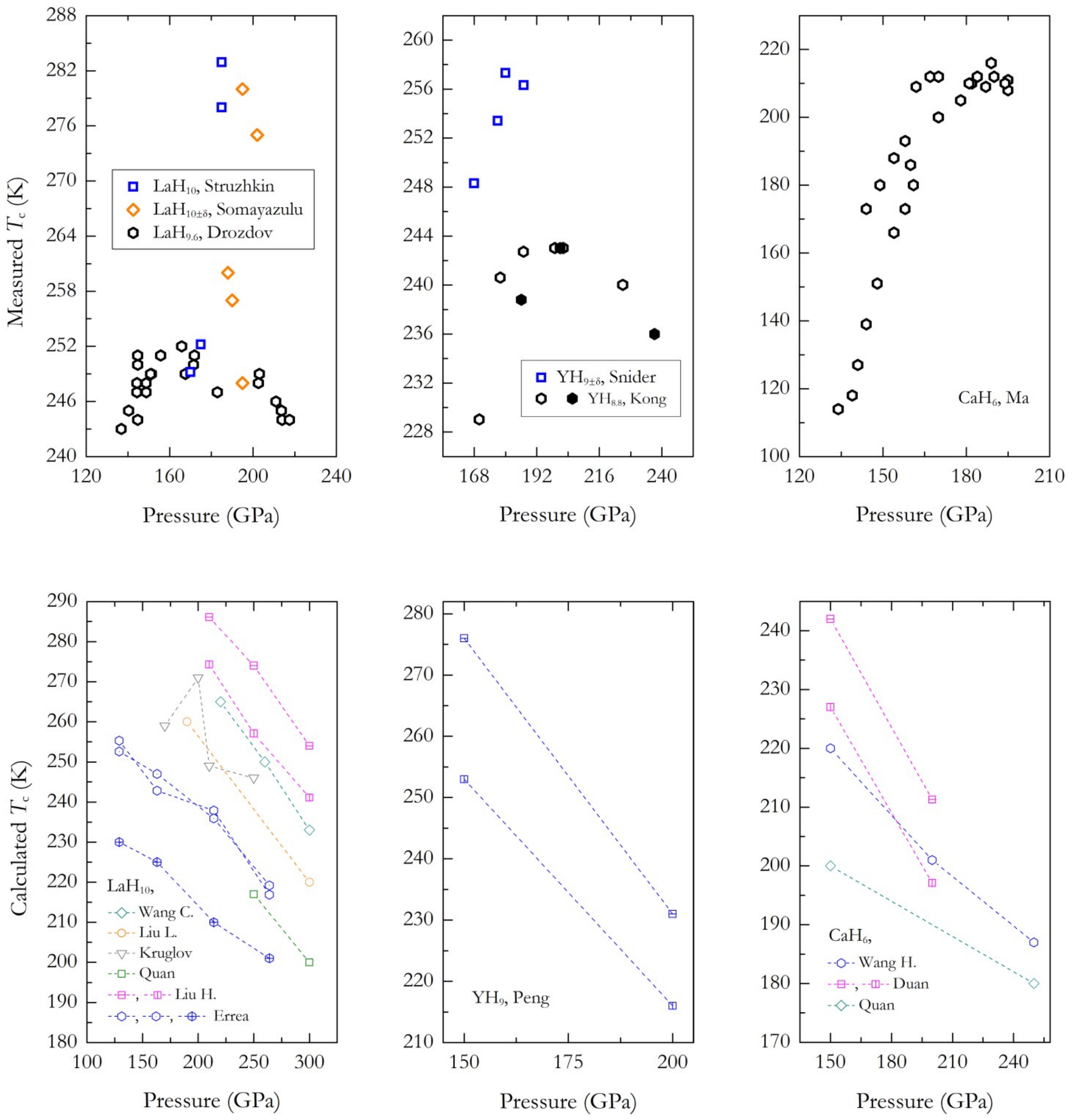

Supplementary Figure 1| The 'positive dT_c/dp contradiction'.

Recent experimental studies by Drozdov *et. al.*[1], Somayazulu *et. al.*[2], Struzhkin *et. al.*[3], Kong *et. al.*[4], Snider *et. al.*[5] and Ma *et. al.*[6] discovered several near-room temperature superconducting polyhydrides, and the measured pressure trend of T_c usually reveals a positive dT_c/dp at some pressure regimes, in contradiction with the negative slope predicted by earlier or subsequent calculations by Peng *et. al.*[7], Liu H. *et. al.*[8], Liu L. *et. al.*[9], Wang C. *et. al.*[10], Quan *et. al.*[11], Errea *et. al.*[12], Kruglov *et. al.*[13], Wang H. *et. al.*[14] and Duan *et. al.*[15, 16] on the same hydride based on BCS theory. The contradiction is clearly seen from the data. The present work focuses on the 250-kelvin lanthanum polyhydride, which has a positive dT_c/dp measured by Drozdov *et. al.* on $LaH_{9.6}$[1], and a negative dT_c/dp calculated by Errea *et. al.*[12] on *fcc*-LaH_{10} at pressures of 137-150 GPa.

Supplementary Figure 2

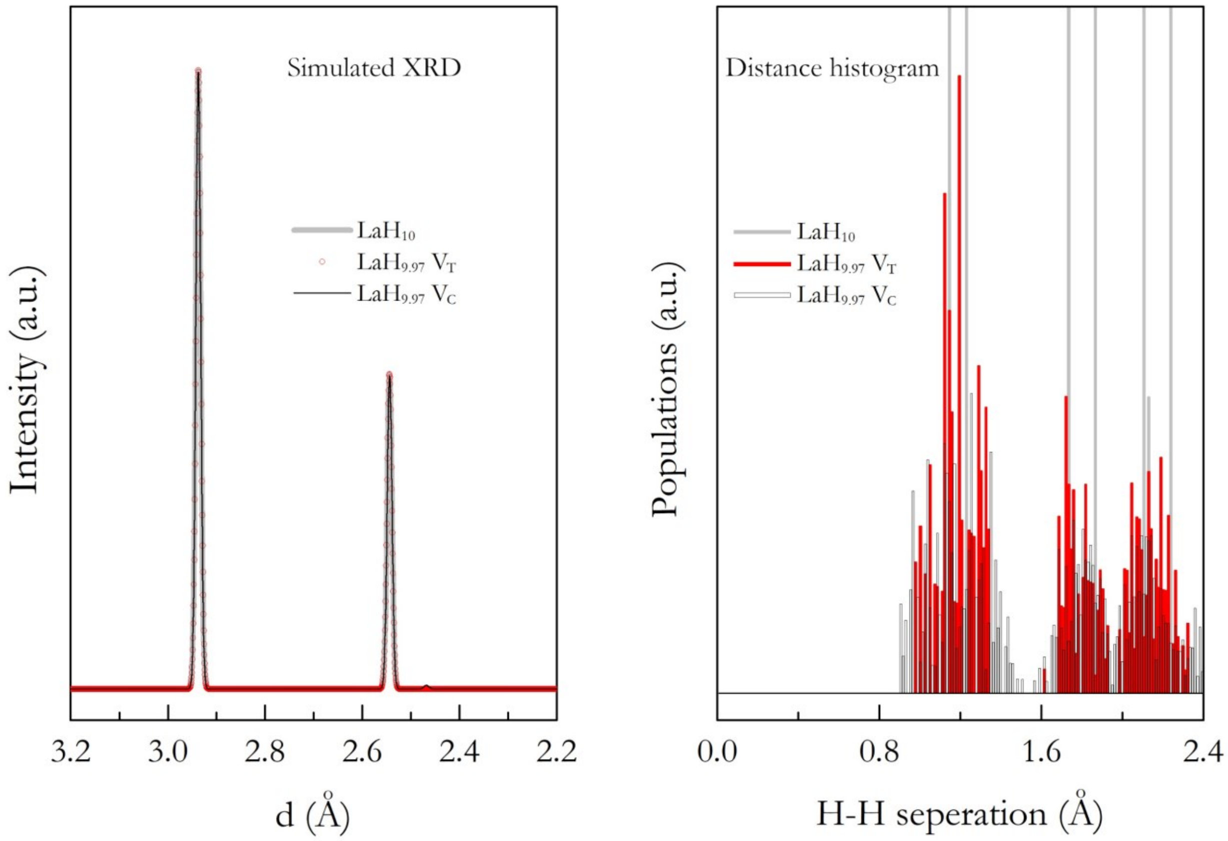

Supplementary Figure 2| Structural features of $LaH_{9.97}$ and *fcc*-LaH_{10} at 0 K.

The simulated XRD patterns of the static V_T and V_C structures of $LaH_{9.97}$ are almost identical to that of the *fcc*-LaH_{10} at 150 GPa, whereas the H-H separations in the V_T and V_C structures of $LaH_{9.97}$ distribute more broadly than that in *fcc*-LaH_{10}, indicating that the lattice distortion spreads out away from a vacancy site in the clathrate H framework of $LaH_{9.97}$. The 'a.u.' is the abbreviation of arbitrary unit, throughout the article.

Supplementary Figure 3

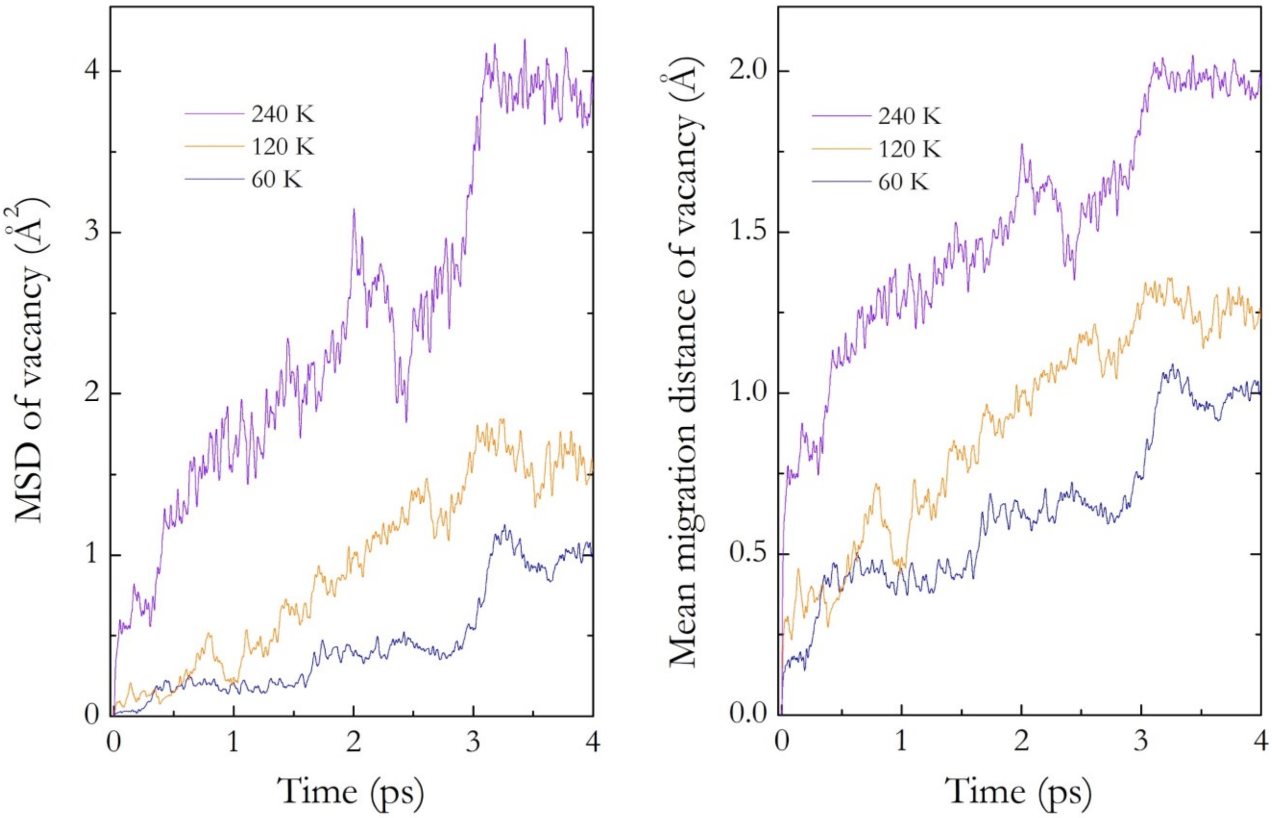

Supplementary Figure 3| Diffusion of vacancy in $LaH_{9.63}$ at 150 GPa.

The vacancy MSDs are derived from proton centroid trajectories in CMD simulations, which show at least one jump over a threshold value of 0.7 Å, the lower boundary of the first peak in the *g*(r), as shown in Supplementary Figure 4. The vacancy mean migration distance is defined as the square root of the MSD.

Supplementary Figure 4

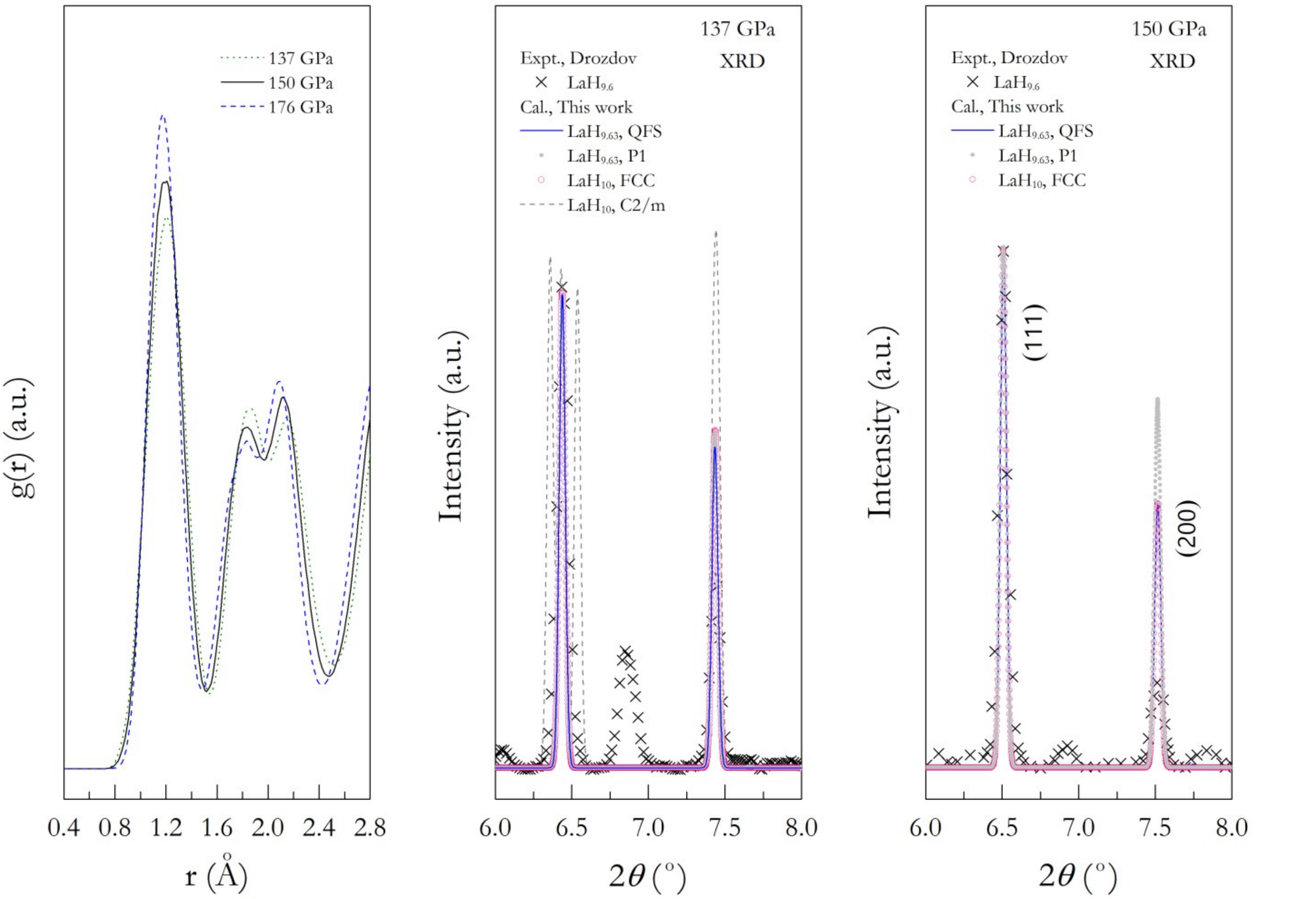

Supplementary Figure 4| Structural features of $LaH_{10-\delta}$.

The pairwise g(r) of H-H extracted from centroid trajectories of CMD simulations at 240 K between 137 and 176 GPa, and a comparison of the XRD pattern measured by Drozdov *et al.*[1] at 137 and 150 GPa to those calculated for the averaged La-substructure in $LaH_{9.63}$, and the static lattices of $P1$-$LaH_{9.63}$, *fcc*-LaH_{10} and $C2/m$-LaH_{10}.

Supplementary Figure 5

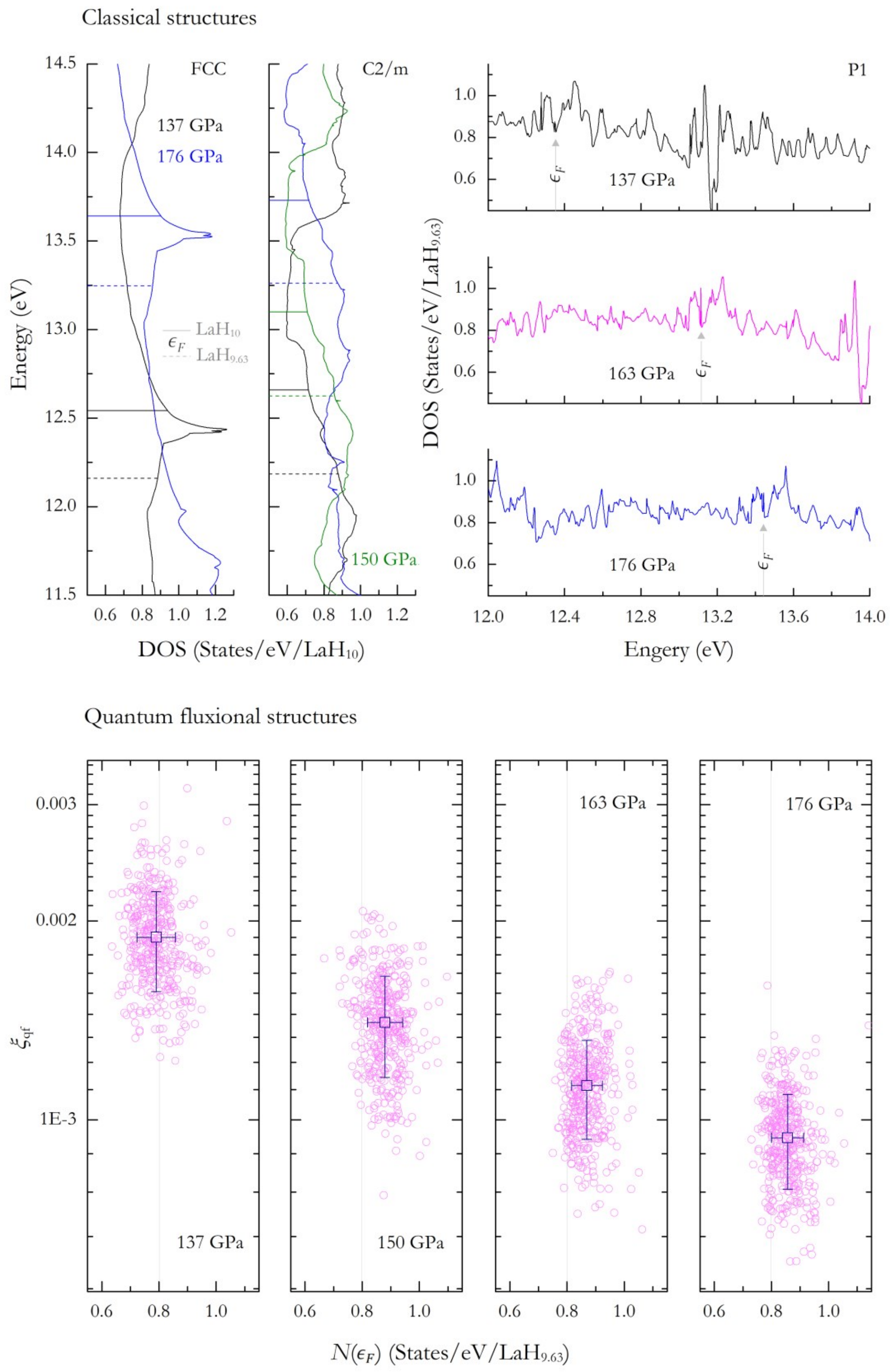

Supplementary Figure 5| Pressure effects on the electronic property of $LaH_{10-\delta}$.

The electron density of states (DOS) for static lattices of *fcc*-LaH_{10}, $C2/m$ -LaH_{10} and $P1$-$LaH_{9.63}$, together with the distribution of $N(\epsilon_F)$ in quantum fluxional $LaH_{9.63}$ at 240 K against the configurational distance ξ_{qf} between 137 ~ 176 GPa, with error bar indicating the standard deviations. In the DOS of static *fcc*- and $C2/m$ -LaH_{10}, the Fermi energy ϵ_F of $LaH_{9.63}$ obtained under the rigid-band approximation is also shown.

Supplementary Figure 6

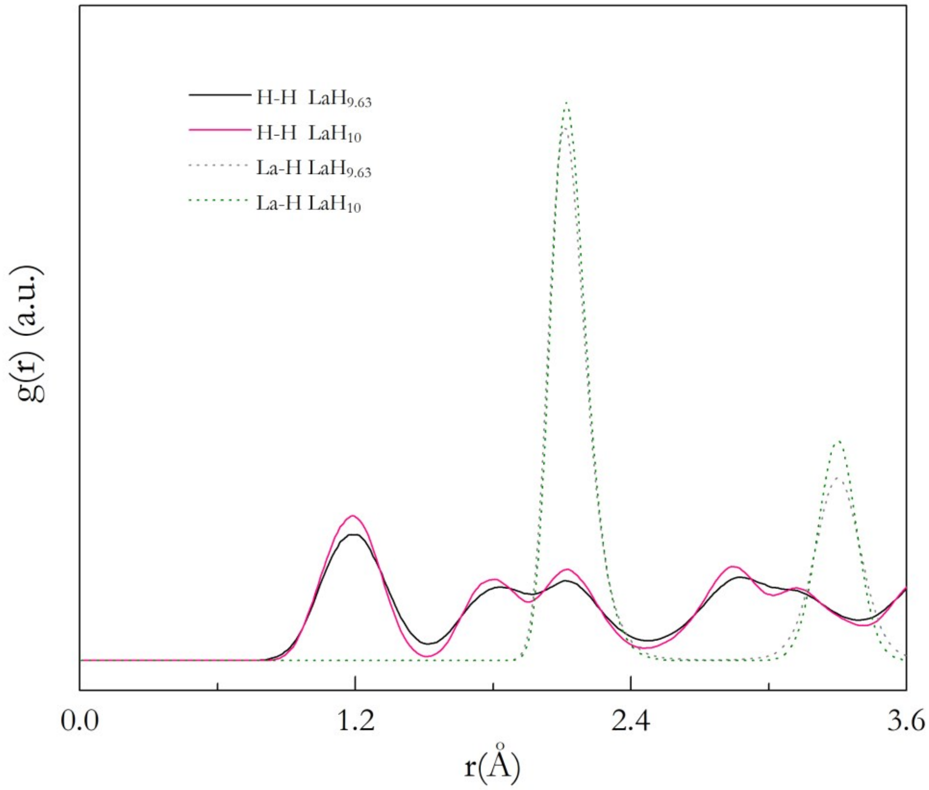

Supplementary Figure 6| Structural similarity between $LaH_{9.63}$ and LaH_{10}.
The pairwise *g*(r) of H-H and La-H extracted from centroid trajectories of CMD simulations at 240 K and 150 GPa for $LaH_{9.63}$ compared to those of *fcc*-LaH_{10}. The similarity in *g*(r) suggest that quantum fluxional $LaH_{9.63}$ largely retains the same local atomic environment as quantum *fcc*-LaH_{10}.

Supplementary Figure 7

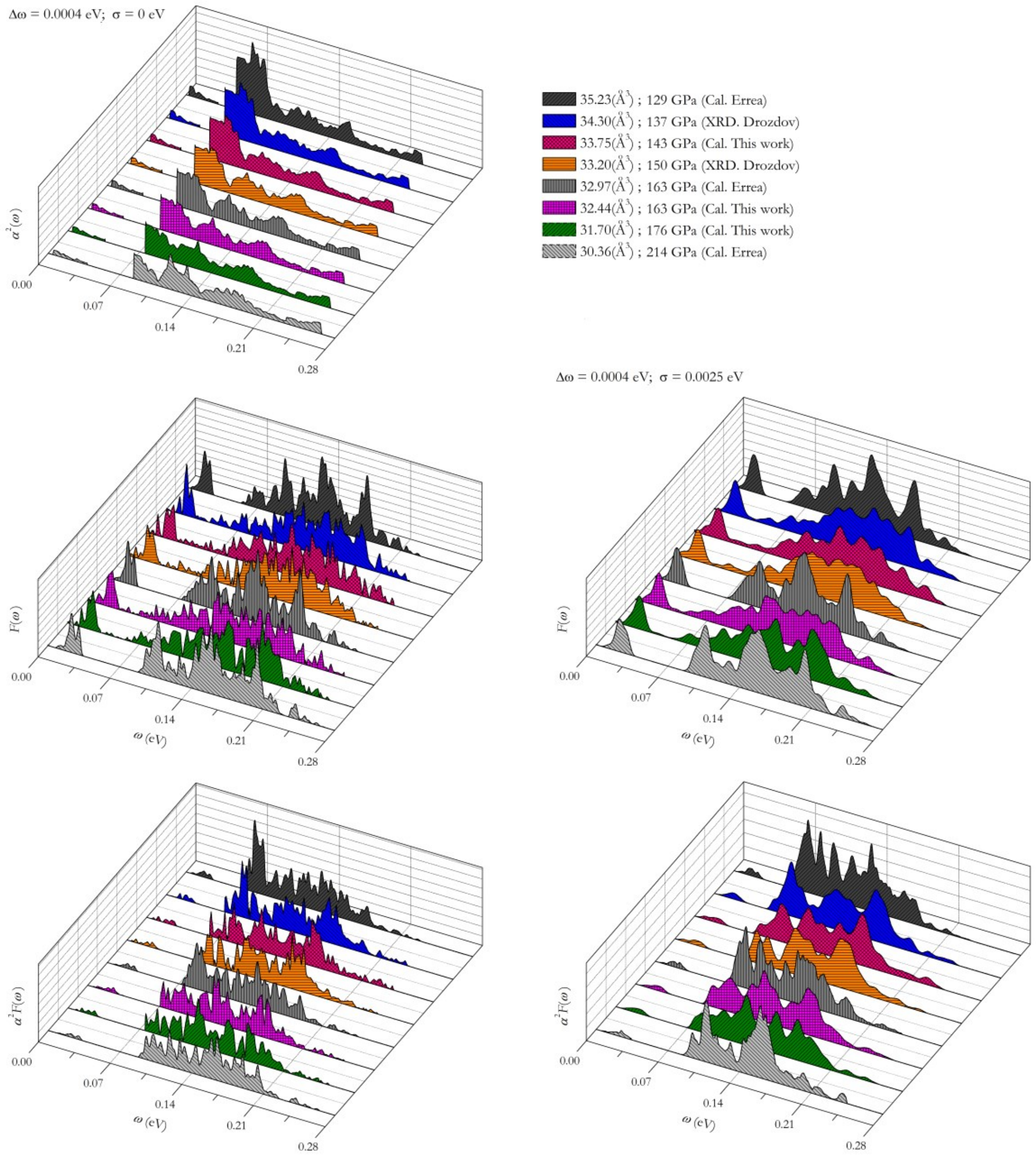

Supplementary Figure 7| The Eliashberg function in $LaH_{10-\delta}$.

The $d\Omega/dp$ (with Ω being ω_{log} or $\overline{\omega}_2$) for estimating the dT_c/dp in quantum fluxional $LaH_{9.63}$ is estimated based on the $F(\omega)$ of $LaH_{9.63}$ obtained by Fourier transforming the velocity autocorrelation functions in the CMD simulations at 240 K in combination with coupling functions $\alpha(\omega)^2$ approximated by that of quantum *fcc*-LaH_{10}[12]. The $\alpha(\omega)^2$ at 129, 163 and 214 GPa are derived from $\alpha(\omega)^2F(\omega)$ and $F(\omega)$ of Ref. 12. The $\alpha(\omega)^2$ at other pressures within 137 ~ 176 GPa are calculated by inverse volume weight interpolation of neighboring coupling functions. With increasing of pressure, $LaH_{9.63}$ exhibits slight phonon hardening in $F(\omega)$, similar to quantum *fcc*-LaH_{10}. Compared to the $F(\omega)$ of quantum *fcc*-LaH_{10}, those of $LaH_{9.63}$ spread a bit wider, which is attributed to temperature effects: The former and the latter are derived at 0 and 240 K, respectively. Smeared $F(\omega)$ is generally adopted to converge EPC parameters, and we smear the $F(\omega)$ by a Gaussian profile with widths (σ) of 0.002 ~ 0.003 eV in this work. The original spectrum and those with σ of 0.0025 eV are shown here. The pressure-volume relations measured by Drozdov *et al.*[1], calculated by Errea *et al.*[12] and estimated in this work are shown in the labels.

Supplementary Table 1

Hydrides	P (GPa)	ΔP (GPa)	T_c (K)	$\Delta T_c/\Delta P$ (K/GPa)	ω_{log} (meV)	$\Delta\omega_{log}/\Delta P$ (meV/GPa; %)	λ	$\Delta\lambda/\Delta P$ (GPa^{-1}; %)	ζ ($1/eV^3$)	$\Delta\zeta/\Delta P$ ($1/eV^3$/GPa; %)	β (eV^3)	$\Delta\beta/\Delta P$ (eV^3/Megabar; %)
**Theoretical analysis of literature data**												
**LaH_{10}** (Ref. 12)	129 ~ 214	85	252.6 ~ 235.9	**-0.196**	76.4 ~ 115.5	**+0.460; +51**	3.62 ~ 2.06	**-0.0184; -43**	93.5 ~ 43.8	**-0.585; -53**	0.039 ~ 0.047	**+0.00941; +21**
**LaH_{10}** (Ref. 8)	210 ~ 300	90	246.0 ~ 223.0	**-0.256**	72.5 ~ 123.2	**+0.563; +70**	3.62 ~ 1.81	**-0.0201; -50**	83.1 ~ 36.1	**-0.522; -57**	0.044 ~ 0.050	**+0.00667; +14**
**LaH_{10}** (Ref. 10)	220 ~ 300	80	234.2 ~ 215.7	**-0.231**	57.9 ~ 115.6	**+0.722; +100**	4.24 ~ 1.86	**-0.0298; -106**	100.1 ~ 40.1	**-0.750; -60**	0.042 ~ 0.046	**+0.00500; +10**
**LaH_{10}** (Ref. 11)	250 ~ 300	50	206 ~ 189	**-0.340**	92 ~ 115	**+0.460; +25**	2.46 ~ 1.80	**-0.0132; -27**	59.1 ~ 40.9	**-0.364; -31**	0.042 ~ 0.044	**+0.00400; +5**
**CaH_6** (Ref. 14)	150 ~ 250	100	221.6 ~ 190.8	**-0.308**	88.8 ~ 118.0	**+0.292; +33**	2.66 ~ 1.63	**-0.0103; -39**	31.9 ~ 17.1	**-0.148; -46**	0.083 ~ 0.095	**+0.01200; +14**
**CaH_6** (Ref. 11)	150 ~ 250	100	200 ~ 180	**-0.200**	90 ~ 117	**+0.270; +30**	2.53 ~ 1.69	**-0.0084; -33**	30.8 ~16.9	**-0.139; -45**	0.082 ~ 0.100	**+0.01800; +21**
**This work**												
**$LaH_{9.63}$**	137 ~ 163	26	209.88 ~ 228.09	**+0.700**	84.20 ~ 94.40	**+0.392; +12**	2.535 ~ 2.452	**-0.0032; -3**	62.887 ~ 56.337	**-0.252; -10**	0.04031 ~ 0.04352	**+0.01235; +8**
	163~ 176	13	228.09 ~ 222.02	**-0.467**	94.40 ~ 99.42	**+0.386; +5**	2.452 ~ 2.240	**-0.0163; -9**	56.337 ~ 50.036	**-0.485; -11**	0.04352 ~ 0.04476	**+0.00954; +3**
	**Stability of the +$\Delta T_c/\Delta P$ slop against variation of β (± 50%)**											
	137 ~ 163	26	**103.06 ~ 110.51**	**+0.287**	84.20 ~ 94.40	**+0.392; +12**	**1.267 ~ 1.226**	**-0.0016; -3**	62.887 ~ 56.337	**-0.252; -10**	**0.02015 ~ 0.02176**	**+0.01235; +8**
	137 ~ 163	26	**290.04 ~ 315.56**	**+0.982**	84.20 ~ 94.40	**+0.392; +12**	**3.802 ~ 3.678**	**-0.0048; -3**	62.887 ~ 56.337	**-0.252; -10**	**0.06046 ~ 0.06528**	**+0.01235; +8**
	**Limit of stability of the +$\Delta T_c/\Delta P$ slop against $\Delta\beta/\Delta P$ slop**											
	137 ~ 163	26	**228.09** ~ 228.09	**-0.00**	84.20 ~ 94.40	**+0.392; +12**	**2.801** ~ 2.452	**-0.0134; -12**	62.887 ~ 56.337	**-0.252; -10**	**0.04454** ~0.04352	**-0.00392; -3**

Supplementary Table 1| Parameters impacting the pressure trend of T_c.

Based on retrievable $\Omega(p)$ (where Ω represents ω_{log} or $\overline{\omega}_2$) (Ref 11) or $\alpha(\omega)^2F(\omega)$ from the literature (Ref 12, 8, 10 and 14), we derived $d\Omega/dp$ and analyzed the role played by several parameters in the pressure trend of T_c using AD equations, with the Coulomb coupling constant μ* set to 0.1. The resulting pressure trends of T_c for cubic LaH_{10} and CaH_6 are in good agreement with the literature (Supplementary Figure 1). The analysis demonstrates that ζ decreases more than two times faster than β increases upon compression, which leads to a negative sign of the $d\lambda/dp$ slope. This suggests that $d\zeta/dp$ dominates the pressure trend of λ. λ has a monotonously negative pressure dependence, but decreases less significantly than ω_{log} increases. However, due to the nonlinear dependence of T_c on λ, either through the well-known exponential function or the 'strong-coupling correction' and 'shape correction' factors (see details in page 14), $d\lambda/dp$ plays a dominant role in determining the dT_c/dp slope.

Our results for $LaH_{9.63}$ are also shown in order to enable a direct comparison. Our $d\omega_{log}/dp$ is comparable to those of cubic LaH_{10} (*fcc*) and CaH_6 (*bcc*), but $d\zeta/dp$ decreases significantly at lower pressures, which reverses the sign of the dT_c/dp slope through a considerable reduction of $d\lambda/dp$ below 163 GPa. We further estimated the limit of stability of the +dT_c/dp slope against variations of β and $d\beta/dp$: I) at a fixed $d\beta/dp$ of 0.01235 eV^3/megabar, decreasing (increasing) β by 50 % results in a decrease (an increase) of dT_c/dp by 59% (41%), but does not change the positive sign; II) at a fixed β of 0.04352 eV^3 at 176 GPa, an about 127 % decrease of the $d\beta/dp$ slope is needed to invert the sign of dT_c/dp. Literature data generally report a positive $d\beta/dp$ slope for cubic hydrides covering a range of H/M ratios (M= La and Ca) from 10 to 6. Therefore, $LaH_{9.63}$ is very unlikely to exhibit a -$d\beta/dp$ slop in qualitative contradiction with most other hydrides. The analysis indicates that the +dT_c/dp slope is robust to errors induced by the approximation of β and $d\beta/dp$ in $LaH_{9.63}$ by those in quantum *fcc*-LaH_{10}.

Supplementary Table 2

Pressure (GPa)	$N(\epsilon_F)$ (1/eV^2)	ω_{log} (meV)	$\bar{\omega}_2$ (meV)	ζ (1/eV^3)	β (eV^3)	λ	T_c (K)
σ =0 for $\alpha(\omega)^2$; σ =0.0025 eV for $F(\omega)$; $\Delta\omega$ = 0.0004 eV							
137	0.79	84.20	112.11	62.887	0.04031	2.535	209.88
143	0.855	84.70	118.26	61.121	0.04128	2.523	212.42
150	0.880	88.24	123.06	58.088	0.04224	2.454	215.94
163	0.869	94.40	124.20	56.337	0.04352	2.452	228.09
176	0.856	99.42	130.82	50.036	0.04476	2.240	222.02
σ = 0.01 & 0.05 eV for $\alpha(\omega)^2$; σ =0.0025 eV for $F(\omega)$; $\Delta\omega$ = 0.0004 eV							
137	0.790	84.16 & 63.11	112.30 & 106.98	62.684 & 69.06	0.04031	2.527 & 2.783	209.30 & 178.21
163	0.869	95.69 & 80.10	124.83 & 121.62	55.769 & 58.75	0.04352	2.427 & 2.557	228.79 & 206.31
176	0.856	100.81 & 88.75	131.68 & 130.54	49.381 & 50.25	0.04476	2.210 & 2.249	222.20 & 202.66
Reshaping $\alpha^2F(\omega)$ by artificial scaling of $\Delta\omega$ to 0.0003 & 0.0005 eV; σ =0 for $\alpha(\omega)^2$; σ = 0.0025 eV for $F(\omega)$							
137	0.790	61.89 & 106.33	83.67 & 140.50	112.900 & 40.042	0.04031	4.551 & 1.614	245.34 & 172.95
163	0.869	69.91 & 119.01	92.90 & 155.53	100.696 & 35.924	0.04352	4.382 & 1.563	267.35 & 186.79
176	0.856	73.56 & 125.12	97.81 & 163.78	89.500 & 31.923	0.04476	4.006 & 1.429	263.36 & 177.40

Supplementary Table 2| Parameters for estimating the pressure trend of T_c.

Parameters for calculating the pressure trend of T_c shown in Fig 4b with smearing σ = 0.0025 eV for $F(\omega)$ are listed here for illustration. Furthermore, we compare the pressure trends of T_c calculated for various σ of $\alpha(\omega)^2$ (*i.e.* 0, 0.01 and 0.05). It is found that the trend is determined by the overall shape of $\alpha(\omega)^2$. Due to the inexistence of a method that can directly calculate the $\alpha^2F(\omega)$ of a quantum fluxional structure, we estimated frequency moments Ω of $\alpha^2F(\omega)$ in $LaH_{9.63}$ based on the calculated $F(\omega)$ of $LaH_{9.63}$ and the $\alpha(\omega)^2$ approximated by that of quantum *fcc*-LaH_{10}[12]. To evaluate the potential errors associated with the approximation, we calculated the T_c using reshaped $\alpha^2F(\omega)$ obtained by artificial scaling of $\Delta\omega$. Empirically, EPC weights the phonon spectrum to lower frequencies in high-T_c hydrides. This weighting effect changes smoothly with pressure, as observed from the shape of $\alpha(\omega)^2$ in quantum *fcc*-LaH_{10} (Supplementary Figure 7). Considering this, and that quantum $LaH_{9.63}$ and *fcc*-LaH_{10} exhibit a similar phonon hardening trend with increasing pressure as well as similar shapes of $F(\omega)$, our results suggest that the pressure trend of T_c is robust to variations of Ω. This indicates that it is practicable to estimate qualitatively the sign of dT_c/dp slop in $LaH_{9.63}$ under this approximation.

Computational Details

Molecular dynamics calculations

Quantum nuclear dynamics were studied using path-integral molecular dynamics (PIMD) and centroid molecular dynamics (CMD), with the massive Nosé-Hoover chain (NHC) thermostats on NVT and NVE ensembles (N-number of particles; V-volume; T-temperature, and E-energy), respectively, as implemented in the PIMD code[17]. NpT-PIMD (p-pressure) simulations were performed to estimate the quantum pressure-volume relations. Beads number was set to 16 for the cases without a specification, and time step was set to 0.5 and 0.05 fs for PIMD and CMD, respectively. A short PIMD simulation was always carried out on the initial structure to prepare the pre-equilibrium state in order to promote the efficiency and stability of the CMD simulation. The parameters of the standard *ab initio* MD simulations are the same as those of the PIMD simulations, except for a reduced bead number of 1. The MD runs of $LaH_{9.63}$ were initialized from different centroid configurations of the 4-picosecond CMD trajectory with a sampling interval of 0.25 ps for 16 short runs of 12000 steps and of 1 ps for 4 long runs of 52000 steps.

The underlying *ab initio* total energy and forces were calculated based on the plane-wave pseudopotential method, as implemented in the Vienna *ab initio* simulation program (VASP)[18]. The convergence criterion for the total energy (Ecc) was chosen to be 3×10^{-6} eV/atom with a plane-wave cutoff of 325 eV and Γ-point sampling of the first Brillouin zone. Decreasing Ecc to 3×10^{-7} eV/atom (run 2) results in a nearly negligible change of the proton diffusion coefficient *D* in $LaH_{9.63}$ at 150 GPa (see Fig. 3a in the main text). The volume of the cubic simulation cells of $LaH_{9.63}$ were fixed at 1097.70, 1079.98, 1062.39, 1038.16, and 1014.35 $Å^3$. The resulting temperature fluctuations at equilibrium are within ± 15 K, ± 10 K and ± 25 K with respect to the target in the PIMD, CMD and MD simulations, respectively. Other details are summarized in Supplementary Table 3.

Supplementary Table 3. Details of simulations.

Phase	Number of Atoms, H : La	Pressures, GPa	Temperature, K	Method	Steps	runs
$LaH_{9.63}$	308:32	150	60, 120, 240	MD	12000	1-16
		137, 143, 150, 163,176	240	MD	52000	1-4
		150	240	PIMD	2000	1,2
				CMD	82000	1,2
		137, 143, 163,176	240	PIMD	2000	1
				CMD	82000	1
		150	60	PIMD	8000	3§
			60, 120	PIMD	2000	1
				CMD	82000	1
$LaH_{9.0}$	288:32	130, 137, 150, 170, 180	300	PIMD†	2000	1
LaH_{10}	320:32	130, 137, 150, 170, 180	300	PIMD†	2000	1
		137, 150	240	PIMD	2000	1
				CMD	42000	1

§NVT simulations with 16, 32 and 64 beads were performed to check the impact of bead number on MSD; †NpT simulations.

The quantum pressure-volume relation of *fcc*-LaH_{10} and *fcc*-LaH_9.

The quantum pressure-volume relation of *fcc*-LaH_{10} and *fcc*-LaH_9 at 300 K was estimated from the volume averaged over 1 ps at equilibrium obtained in NpT-PIMD simulations using a cubic box and Martyna's equation of motion, as implemented in the PIMD code[17]. Our results reveal a H/La ratio of 9.54 (or 9.71) for the experimental samples synthesized at 150 GPa (or 137 GPa), in good agreement with experimental estimation of a $LaH_{9.6}$[1]. To ensure our pressure trend of T_c can directly compare with the experimental one, we built structural models at 137 and 150 GPa using experimental lattice parameters. This guarantees that the coincidence of this pressure range (*i.e.* '137-150 GPa') between calculation and experiments does not dependent on the experimental or theoretical pressure scales. Notably, this is indeed the pressure range that the positive dT_c/dp slope were measured experimentally and calculated in this work. The PBE functionals may lead to potential shift of the calculated T_c *v.s.* pressure curve in relative to the experimental measured one at pressure above 150 GPa, however, which does not affect our conclusion. The quantum pressure of 143, 163 and 176 GPa were estimated based on interpolation.

The mean square displacement, $\langle \Delta \boldsymbol{r}^2 \rangle$

$$\langle \Delta r(t)^2 \rangle = \langle |r_i(t) - r_0(t) - [r_{cm}(t) - r_{cm}(0)]|^2 \rangle_I,$$

where $r_i(t)$ is the position of the diffusive proton *i*, which exhibits at least one jump over a threshold value of 0.7 Å over the course of the simulation, $r_{cm}(t)$ represents the position of the center of mass of the system at the time *t*, and the $\langle\ \rangle$ represent an average over 10 time steps and the total number of 'particle' *I*, which equals 308 for proton and 12 for vacancy in $LaH_{9.63}$. In the simulations, some of diffusive protons hop back to the previously occupied position occasionally due to 'traffic jam', which results in a decrease of $\langle \Delta \boldsymbol{r}^2 \rangle$. The proton traffic jam and local structural relaxation result in plateaus in $\langle \Delta \boldsymbol{r}^2 \rangle$ curves in both the CMD and MD simulations. However, $\langle \Delta \boldsymbol{r}^2 \rangle$ generally increases with increasing of t, which is clearly revealed by long-time MD simulations of 24 ps.

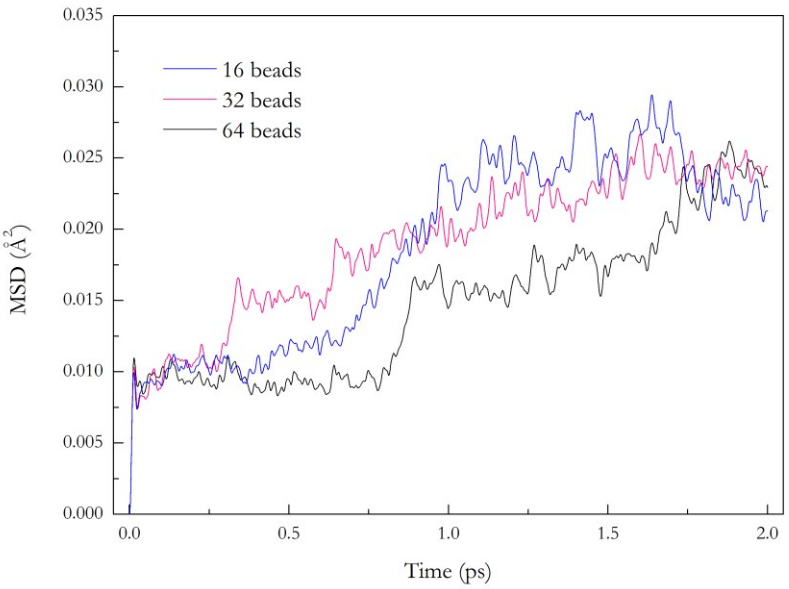

Supplementary Figure 8. MSD of PIMD simulation with various bead numbers at 60 K.

MSDs derived from the centroid trajectory of PIMD simulations at 150 GPa and an equilibrium temperature of 60 K with 16, 32 and 64 beads are shown in Supplementary Figure 8. All curves suggest quantum proton diffusion at this temperature. Considering that the strength of NQE increases with decreasing temperature, larger bead number gives better MSD. The difference of $\langle \Delta \boldsymbol{r}^2 \rangle$ values at the end of the simulation (*i.e.* 2 ps) is not significant, which may be attributed to the 'traffic jam' of protons in $LaH_{9.63}$ associated with the small amount of stoichiometric defect.

The diffusion coefficient, *D*

We approximate the proton diffusion coefficient D in $LaH_{9.63}$ from the slope of $\langle \Delta r^2 \rangle$ obtained from a linear fit, as shown in Supplementary Figure 9 for CMD simulations run1 and run2 at 150 GPa and 240 K. D is computed from $D = slope(\langle \Delta r^2 \rangle)/6$.

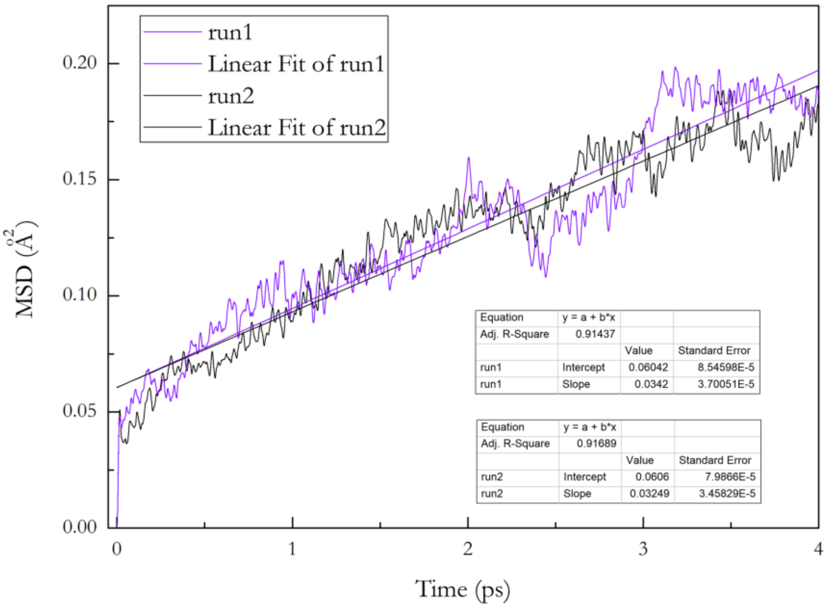

Supplementary Figure 9. Linear fit of MSD.

Vacancy formation enthalpy, H^f

We carried out a full variable-cell optimization (using ISIF=3 in VASP) of *fcc*-LaH_{10}[7, 8] (in a 2×2×2 supercell of the conventional unit cell containing 352 atoms) and *B*2/*n*-H_2 (in the conventional unit cell of 48 atoms)[19] at pressures between 120 and 220 GPa with an interval of 5 GPa. We employed *k*-mesh grids of sizes 3×3×3 and 15×9×9, respectively, to carry out the required DFT calculations.

The V_T and V_C $LaH_{9.97}$ vacancy structures were then constructed by removing the appropriate H atom from the relaxed *fcc*-LaH_{10} structure at each pressure, as described in the main text. Variable-cell optimization of these structures induced proton diffusion, resulting in a distortion of the structures. In order to isolate the effect of the vacancies, therefore, we only relaxed the atomic positions, keeping the cell shape and volume fixed (ISIF=2 in VASP). We used a *k*-mesh grid equivalent to that for *fcc*-LaH_{10}. A cut-off energy of 325 eV and a convergence criterion for the total energy of 3×10^{-7} eV/atom were employed in all calculations.

Using the resulting *V-E* relationship of the relaxed structures, the pressure-enthalpy curves were then calculated using the third-order Birch–Murnaghan isothermal equation of state[20]. From the enthalpies for each pressure, the H^f of the V_C and V_T structures were calculated as follows,

$$H^f = H(LaH_{9.97}) - H(fcc\text{-}LaH_{10}) + H(B2/n\text{-}H_2)/48,$$

with the configurationally averaged H^f expressed as,

$$H^f(\text{average}) = 0.2\times H^f(V_T) + 0.8\times H^f(V_C).$$

The calculated pressure-volume curve gives a pressure shift of ~5 GPa for $LaH_{9.97}$ with respect to the experimental measurements on *fcc*-$LaH_{9.6}$ at 120-220 GPa, which does not affect our discussions.

It should be noted that H^f is calculated classically in the static lattice approximation on the Born–Oppenheimer energy surface, which differs from the configurational energy surface of the quantum crystal (e.g., *fcc*-LaH_{10} in Ref. 12) or the free energy surface of the classical or quantum crystal at finite temperature. The stability and pressure boundary of the

vacancy structure may be affected by more complex physical effects existing in the superconducting samples, for instance nuclear quantum effects (including zero-point motion of nuclei and proton tunnelling), or temperature effects. Indeed, temperature effects only enhance the formation of vacancies. In this sense, the conclusion from the H^f curves shown in Fig.1a of the main text, namely that vacancy formation is favored below 158 GPa in the classical crystal picture, while being based on a model calculation subject to many approximations, indicates that lanthanum superhydride should be rather prone to vacancy formation.

The configurational distance, ξ

The crystal fingerprint technique utilized in this study is based on Gaussian overlap matrices, which represent the local environments of all atoms in a unit cell and can efficiently determine configurational distances ξ between crystalline structures satisfying the mathematical requirements of a metric[21]. The ξ of the QFS of $LaH_{9.63}$ *vs.* various static structures (e.g. *fcc*, *C*2/*m* and *P*1) were obtained by statistical analysis over the ξ of 400 centroid configurations sampled from 16-bead CMD trajectories of 4 ps with a time interval of 10 fs at each pressure.

Electronic density of states at the Fermi level, $N(\epsilon_F)$

The $N(\epsilon_F)$ is of central importance to the understanding of conventional superconductivity for hydrogen-rich materials. It is quite general that the pressure dependence of $N(\epsilon_F)$ directly correlates to the pressure trend of the superconducting T_c. The $N(\epsilon_F)$ of the quantum structures of $LaH_{9.63}$ was calculated by carrying out a statistical average over the $N(\epsilon_F)$ of 400 centroid configurations sampled from 16-bead CMD trajectories of 4 ps with a time interval of 10 fs at each pressure. The $N(\epsilon_F)$ of each centroid configuration was calculated using the tetrahedron method with Blöchl corrections[22], with a convergence criterion for the total energy of 3×10^{-6} eV/atom for a cut-off energy of 325 eV and a 5×5×5 *k*-mesh grid. The $N(\epsilon_F)$ of static *fcc*-LaH_{10}, *C*2/*m*-LaH_{10}, and *P*1-LaH_{10} were calculated based on primitive cells by the tetrahedron method with Blöchl corrections, with a convergence criterion for the total energy of 3×10^{-6} eV/atom for a cut-off energy of 325 eV and 21×21×21, 27×27×15 and 5×5×5 *k*-mesh grids, respectively. For the *fcc* and *C*2/*m* phases, the $N(\epsilon_F)$ of static $LaH_{9.63}$ were estimated based on the rigid-band model[23] of the electronic structure of LaH_{10} by virtue of an artificial shift of ϵ_F.

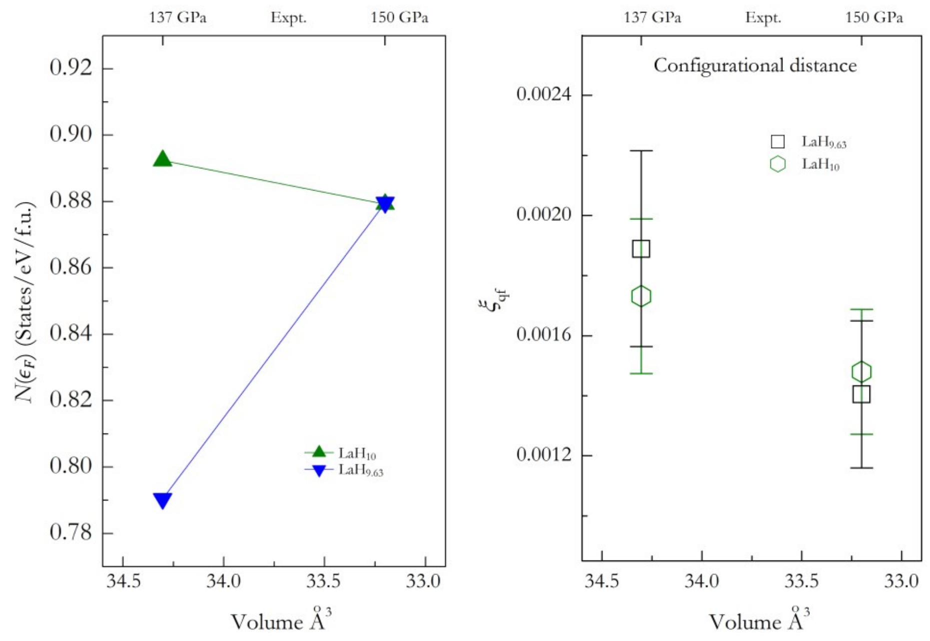

Supplementary Figure 10. The $N(\epsilon_F)$ and ξ (of H substructure) in $LaH_{9.63}$ compared to those of LaH_{10}. The f.u. is the abbreviation of formula unit, throughout the article.

Comparison on $N(\epsilon_F)$ and ξ between $LaH_{9.6}$ and LaH_{10}

As shown in Supplementary Figure 10, we compared the configurationally averaged ξ and $N(\epsilon_F)$ in quantum $LaH_{9.63}$ and LaH_{10} in the pressure range of 137–150 GPa. The results suggest that the H sublattice distortion is suppressed in the vacancy-free case. Moreover, a negative $dN(\epsilon_F)/dp$ slop is observed in LaH_{10}, in contrast to the positive $dN(\epsilon_F)/dp$ slop of $LaH_{9.63}$.

The frequency moments of $\alpha^2F(\omega)$ in $LaH_{9.63}$, ω_{log} and $\bar{\omega}_2$

The $\alpha^2F(\omega)$ of $LaH_{9.63}$ is estimated based on the $F(\omega)$ of $LaH_{9.63}$ derived by Fourier transforming the velocity autocorrelation functions (VACF) in the CMD simulations at 240 K in combination with coupling functions $\alpha(\omega)^2$ approximated by that of quantum *fcc*-LaH_{10}:

1. Deriving the $F(\omega)$ of $LaH_{9.63}$ by Fourier transforming the VACF in the CMD simulations at 240 K and volume (V) of 34.30, 33.75, 33.20, 32.44, and 31.70 $Å^3$/f.u.;
2. Calculating the $\alpha(\omega)^2$ from $\alpha^2F(\omega)$ and $F(\omega)$ in quantum *fcc*-LaH_{10}: $\alpha(\omega)^2 = \alpha^2F(\omega)/F(\omega)$ at volume (V') of 35.23, 32.97 and 30.36 $Å^3$/f.u., and deriving the $\alpha(\omega)^2$ at V by inverse volume weight interpolation of $\alpha(\omega)^2$ of neighboring V';
3. Calculating the $\alpha(\omega)^2F(\omega)$ of $LaH_{9.63}$ based on $F(\omega)$ of $LaH_{9.63}$ and $\alpha(\omega)^2$ of quantum *fcc*-LaH_{10}: $\alpha^2F(\omega)_{\_LaH9.63} = \alpha(\omega)^2{}_{\_LaH10} \times F(\omega)_{\_LaH9.63}$ at various V values;
4. Calculating the ω_{log} and $\bar{\omega}_2$ following their definitions in Ref. 24.

The frequency step $\Delta\omega$ is set to 0.0004 eV in the calculations. To include the vibrational modes in both of low- and high-frequency regime, the $F(\omega)$ are derived from the VACFs of the La and H sublattices separately. Since smeared $F(\omega)$ is generally adopted to converge EPC parameters, we smear $F(\omega)$ with Gaussian profiles with widths (σ) of 0.002 ~ 0.003 eV. ω_{log} and $\bar{\omega}_2$ are therefore obtained following their definitions in Ref. 24. In addition, we reshaped the $\alpha^2F(\omega)$ of $LaH_{9.63}$ by artificial scaling of $\Delta\omega$ to 0.0003 and 0.0005 eV to evaluate the potential errors associated with the approximation in step 3, see the caption of Supplementary Table 2.

The electron-phonon coupling constant, λ

We estimate λ in $LaH_{9.63}$ using McMillan's formula[25] ($\lambda = N(\epsilon_F)\langle I^2\rangle/M\bar{\omega}_2^2$) as follows:

1. Combining variables as $\lambda = \beta \cdot \zeta$ with $\beta = \langle I^2\rangle/M$ and $\zeta = N(\epsilon_F)/\bar{\omega}_2^2$;
2. Estimating $\zeta(V)$ based on $N(\epsilon_F)$ and $\bar{\omega}_2$ of $LaH_{9.63}$ at V of 34.30, 33.75, 33.20, 32.44, and 31.70 $Å^3$/f.u.;
3. Calculating $\beta(V')$ from λ, $N(\epsilon_F)$ and $\bar{\omega}_2$ of quantum *fcc*-LaH_{10} in Ref. 12 at V' of 35.23, 32.97 and 30.36 $Å^3$/f.u. and deriving the $\beta(V)$ by inverse volume weight interpolation of neighboring $\beta(V')$;
4. Calculating $\lambda(V)$ in $LaH_{9.63}$ using the estimated $\zeta(V)$ of $LaH_{9.63}$ and the $\beta(V)$ of quantum *fcc*-LaH_{10}.

Critical temperature of superconductivity, T_c

The T_c was evaluated using the Allen–Dynes-modified McMillan equation (AD)[24],

$$T_c = \frac{f_1 f_2 \omega_{log}}{1.2} exp\left[\frac{-1.04(1+\lambda)}{\lambda-\mu^*(1+0.62\lambda)}\right],$$

where f_1 and f_2 are the 'strong-coupling correction' and 'shape correction' factors for strong-coupling systems:

$$f_1 = [1 + (\lambda/\Lambda_1)^{3/2}]^{1/3} \text{ with } \Lambda_1 = 2.46(1 + 3.8\mu^*);$$

$$f_2 = 1 + (\bar{\omega}_2/\omega_{log} - 1)\lambda^2/(\lambda^2 + \Lambda_2^2) \text{ with } \Lambda_2 = 1.82(1 + 6.3\mu^*)(\bar{\omega}_2/\omega_{log}).$$

μ^* is the Coulomb coupling constant, which is set to a typical value of 0.1 in this wok.

Structural information of static $LaH_{10\text{-}\delta}$.

The simulation cells of *fcc*-LaH_9 (320 atoms) and *fcc*-LaH_{10} (352 atoms) are initialized by a 2×2×2 extension of the unit cell of *F*-43*m*-LaH_9[13] and *Fm*-3*m*-LaH_{10}[12], respectively. We initialize the $LaH_{9.63}$ structure (340 atoms) by adding randomly 20 H atoms to the simulation cell of *fcc*-LaH_9, constraining neighboring vacancies to be no less than 4.4 Å apart to ensure an approximately uniform vacancy distribution, as shown in Supplementary Figure 11. A PIMD simulation (2000 steps) was always carried out on the initial structure to prepare the pre-equilibrium state for the CMD simulation. Vacancy diffusion generally results in a random distribution of vacancies on the 8c and 32f Wyckoff position in *Fm*-3*m*-LaH_{10} within several hundred PIMD steps. The static *P*1-$LaH_{9.63}$, mimicking the quantum fluxional $LaH_{9.63}$, is built by scaling the lattice parameter *a* of a QFS sampled from the CMD simulation of $LaH_{9.63}$ at 176 GPa. The initial vacancy structure of $LaH_{9.63}$ and static *P*1-$LaH_{9.63}$ at 150 GPa can be found at the link—https://doi.org/10.6084/m9.figshare.20484420.v1.

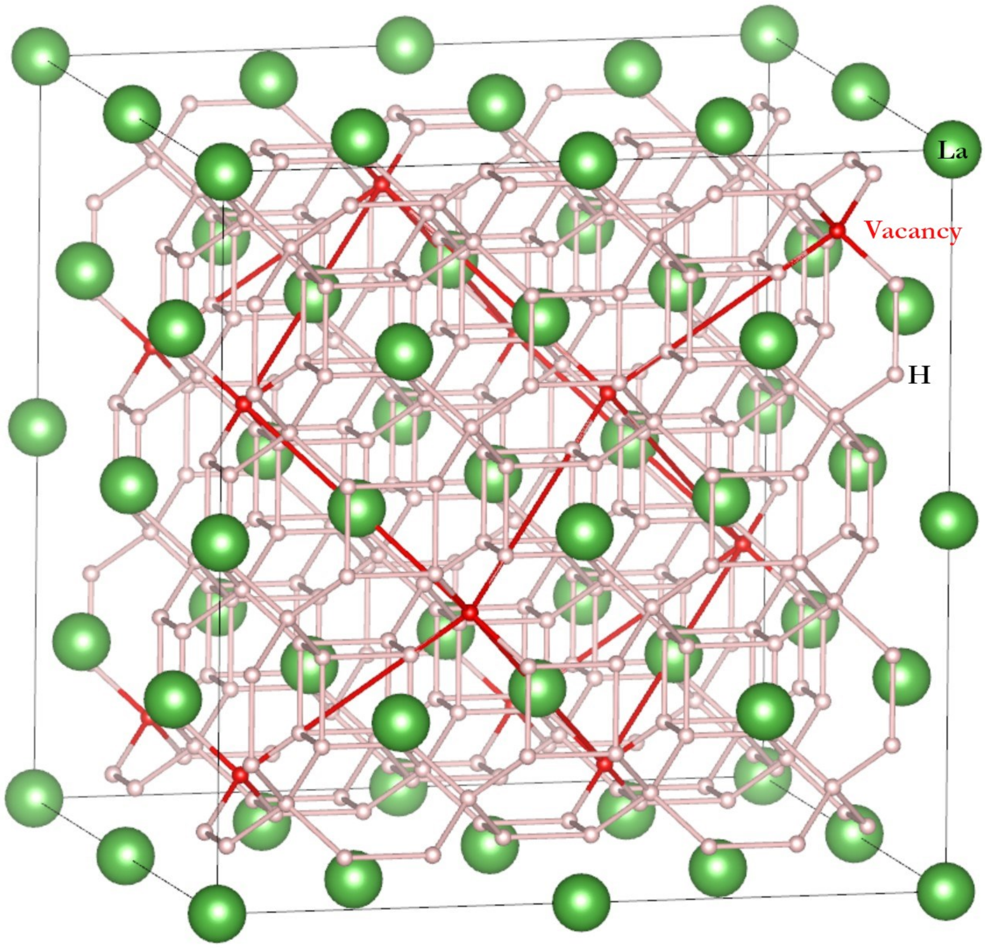

Supplementary Figure 11. Initial vacancy structure of $LaH_{9.63}$.

Supplementary References

(Repeating citations for some references of the main text)

1. Drozdov AP, *et al.* Superconductivity at 250 K in lanthanum hydride under high pressures. *Nature* **569**, 528-531 (2019).

2. Somayazulu M, *et al.* Evidence for Superconductivity above 260 K in Lanthanum Superhydride at Megabar Pressures. *Phys Rev Lett* **122**, 027001 (2019).

3. Struzhkin V, *et al.* Superconductivity in La and Y hydrides: Remaining questions to experiment and theory. *Mat Rad Extr* **5**, 028201 (2020).

4. Kong P, *et al.* Superconductivity up to 243 K in the yttrium-hydrogen system under high pressure. *Nat Commun* **12**, 5075 (2021).

5. Snider E, *et al.* Synthesis of Yttrium Superhydride Superconductor with a Transition Temperature up to 262 K by Catalytic Hydrogenation at High Pressures. *Phys Rev Lett* **126**, 117003 (2021).

6. Ma L, *et al.* High-Temperature Superconducting Phase in Clathrate Calcium Hydride CaH_6 up to 215 K at a Pressure of 172 GPa. *Phys Rev Lett* **128**, 167001 (2022).

7. Peng F, Sun Y, Pickard CJ, Needs RJ, Wu Q, Ma Y. Hydrogen Clathrate Structures in Rare Earth Hydrides at High Pressures: Possible Route to Room-Temperature Superconductivity. *Phys Rev Lett* **119**, 107001 (2017).

8. Liu H, Naumov II, Hoffmann R, Ashcroft NW, Hemley RJ. Potential high-T_c superconducting lanthanum and yttrium hydrides at high pressure. *Proc Natl Acad Sci USA* **114**, 6990 (2017).

9. Liu L, Wang C, Yi S, Kim KW, Kim J, Cho J-H. Microscopic mechanism of room-temperature superconductivity in compressed LaH_{10}. *Phys Rev B* **99**, 140501 (2019).

10. Wang C, Yi S, Cho J-H. Pressure dependence of the superconducting transition temperature of compressed LaH_{10}. *Phys Rev B* **100**, 060502 (2019).

11. Quan Y, Ghosh SS, Pickett WE. Compressed hydrides as metallic hydrogen superconductors. *Phys Rev B* **100**, 184505 (2019).

12. Errea I, *et al.* Quantum crystal structure in the 250-kelvin superconducting lanthanum hydride. *Nature* **578**, 66-69 (2020).

13. Kruglov IA, *et al.* Superconductivity of LaH_{10} and LaH_{16} polyhydrides. *Phys Rev B* **101**, 024508 (2020).

14. Wang H, Tse JS, Tanaka K, Iitaka T, Ma Y. Superconductive sodalite-like clathrate calcium hydride at high pressures. *Proc Natl Acad Sci USA* **109**, 6463 (2012).

15. Xie H, *et al.* High-temperature superconductivity in ternary clathrate $YCaH_{12}$ under high pressures. *J Phys: Condens Matter* **31**, 245404 (2019).

16. Song H, Zhang Z, Cui T, Pickard CJ, Kresin VZ, Duan D. High T_c superconductivity in heavy Rare Earth Hydrides. *Chin Phys Lett* **38**, 107401 (2021).

17. Shiga M, Tachikawa M, Miura S. A unified scheme for ab initio molecular orbital theory and path integral molecular dynamics. *J Chem Phys* **115**, 9149-9159 (2001).

18. Kresse G, Furthmüller J. Efficient iterative schemes for ab initio total-energy calculations using a plane-wave basis set. *Phys Rev B* **54**, 11169-11186 (1996).

19. Pickard CJ, Needs RJ. Structure of phase III of solid hydrogen. *Nat Phys* **3**, 473-476 (2007).

20. Birch F. Finite Elastic Strain of Cubic Crystals. *Phys Rev* **71**, 809-824 (1947).

21. Zhu L, *et al.* A fingerprint based metric for measuring similarities of crystalline structures. *J Chem Phys* **144**, 034203 (2016).

22. Blöchl PE. Projector augmented-wave method. *Phys Rev B* **50**, 17953-17979 (1994).

23. Boeri L. Understanding Novel Superconductors with Ab Initio Calculations. In: *Handbook of Materials Modeling: Applications: Current and Emerging Materials* (eds Andreoni W, Yip S). Springer International Publishing (2018).

24. Allen PB, Dynes RC. Transition temperature of strong-coupled superconductors reanalyzed. *Phys Rev B* **12**, 905-922 (1975).

25. McMillan WL. Transition Temperature of Strong-Coupled Superconductors. *Phys Rev* **167**, 331-344 (1968).
